# Heteroplasmy and Ancient Translocation of Mitochondrial DNA to the Nucleus in the Chinese Horseshoe Bat (*Rhinolophus sinicus*) Complex

**DOI:** 10.1371/journal.pone.0098035

**Published:** 2014-05-19

**Authors:** Xiuguang Mao, Ji Dong, Panyu Hua, Guimei He, Shuyi Zhang, Stephen J. Rossiter

**Affiliations:** 1 Institute of Molecular Ecology and Evolution, Institute for Advanced Studies in Multidisciplinary Science and Technology, East China Normal University, Shanghai, China; 2 School of Biological and Chemical Sciences, Queen Mary University of London, London, United Kingdom; Ben-Gurion University of the Negev, Israel

## Abstract

The utility and reliability of mitochondrial DNA sequences in phylogenetic and phylogeographic studies may be compromised by widespread and undetected nuclear mitochondrial copies (numts) as well as heteroplasmy within individuals. Both numts and heteroplasmy are likely to be common across diverse taxa yet few studies have characterised their frequencies and variation at the intra-specific level. Here we report the presence of both numts and heteroplasmy in the mitochondrial control region of the Chinese horseshoe bat *Rhinolophus sinicus*. In total we generated 123 sequences from 18 bats, which contained two different numt clades (i.e. Numt-1 and Numt-2) and one mtDNA clade. The sequence divergence between Numt-1 and Numt-2 was 16.8% and each numt type was found in all four *R. sinicus* taxa, suggesting either two ancient translocations of mitochondrial DNA into the nucleus from the same source taxon, or a single translocation from different source taxa that occurred before the split of *R. sinicus* into different lineages. Within the mtDNA clade, phylogenetic relationships among the four taxa of *R. sinicus* were similar to those seen in previous results. Based on PCR comparisons, heteroplasmy was inferred between almost all individuals of *R. sinicus* with respect to sequence variation. Consistent with introgression of mtDNA between Central *sinicus* and *septentrionalis*, individuals from these two taxa exhibited similar signatures of repeated sequences in the control region. Our study highlights the importance of testing for the presence of numts and heteroplasmy when applying mtDNA markers to phylogenetic studies.

## Introduction

Mitochondrial DNA (mtDNA) has long been widely used as a source of molecular markers in animal phylogenetic and phylogeographic studies [1], [2] due to its high mutation rate and associated intraspecific polymorphism, general assumed lack of recombination [1], and its high copy number within cells.

However, many mitochondrial sequences have been incorporated into the nuclear genome, and the presence of these so-called nuclear mitochondrial copies (numts, [3]) has been recorded in diverse taxa [4], [5], [6]. Numts can obscure signals from real mtDNA [4], especially when the translocation of mtDNA to the nucleus has occurred recently. In such cases, insufficient time may have lapsed for mutations to accrue via relaxed selection, leading numts to be amplified by primers designed for their mitochondrial counterparts [7], [8]. Indeed, numts can even be amplified more efficiently than authentic mtDNA sequences when using universal primers designed based on sequence comparisons from multiple taxa [9] and thus, direct sequencing of PCR products can lead to ambiguous sequences where numts are co-amplified together with authentic mtDNA. To date several strategies have been applied to check for the presence of numts [10]. Potential nuclear copies of mitochondrial coding segments are often inferred from the presence of frameshift mutations and/or stop codons [11], although these mutations cannot be used to detect nuclear copies of the mitochondrial control region, in which they can occur naturally.

**Figure 1:**
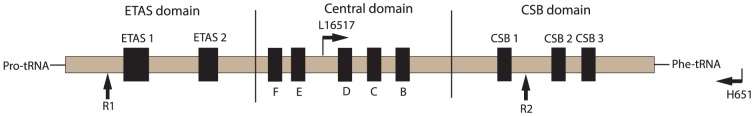
Schematic organization of the bat mitochondrial control region. Two elements in the ETAS domain, five conserved blocks in the Central domain, and two elements in the CSB domain are shown in filled squares. Two repeated sequences (R1 and R2) are located in the ETAS domain and CSB domain, respectively. The locations of the primers used in this study, L16517 and H651, are also shown.

In addition to numts, intra-individual variation in mtDNA sequences can also arise from heteroplasmy, where multiple mitochondrial genomes co-exist within one individual [12], [13]. Heteroplasmy can arise from both sequence variation (i.e. point mutations) and length polymorphisms, the latter of which appears to occur more frequently in natural populations [14], [15], [16]. In most such cases, these heteroplasmic length variations are caused by copy number variation in tandemly repeated sequences located in parts of the mitochondrial DNA control region. The mammalian control region commonly contains three functional domains: the extended terminal associated sequences (ETAS) domain, the central domain (the most conserved part in the control region), and the conserved sequences block (CSB) domain. A long repeated sequence (R1) has been detected in the ETAS domain of the control region in some mammals [17], [18], whereas heteroplasmic length variation has been reported in many species [17], [19], [20] and appears to result from a tandemly repeated array (R2) in the CSB domain (Figure 1). These tandem repeats can arise by DNA slippage during replication, which is thought to commonly generate simple repetitive sequences [21], as well as by mtDNA recombination [22],[23],[24]. Yet to date little is known about the functional importance of heteroplasmic length variation [25].

Despite numerous records of numts in a range of mammals, there have been relatively few reported cases in bats [26], [27], [28], [29], Similarly, heteroplasmy has also only been documented in very few bat species [19], [20], [30], [31], [32], with most previous studies having focused on inter-specific differences. Here we tested for the presence of numts and heteroplasmy in the mitochondrial control region of the Chinese horseshoe bat *Rhinolophus sinicus*. Previously we performed phylogenetic analyses on this species using datasets of two mtDNA protein coding genes and four nuclear genes, which identified four divergent lineages: East *sinicus*, Hainan *sinicus*, Central *sinicus* and *septentrionalis* [33], [34]. Introgression of mtDNA was detected between Central *sinicus* and *septentrionalis* [34]. In our current study we first generate partial sequences of the mitochondrial control region, including the central domain and the CSB domain, from multiple individuals of each of the four lineages. Where we observe putative numts, we attempt to determine the frequency of the inferred transfer events and the timing of these events relative to the divergence within this species complex by incorporating the available full-length mitochondrial control region sequences from other *Rhinolophus* species. Where heteroplasmy is observed, we test the hypothesis that Central *sinicus* and *septentrionalis* could show a similar signature of heteroplasmy due to introgression of mtDNA.

## Materials and Methods

### Ethics Statement

All tissue used in this study were sampled from bats for our former project [34]. The non-lethal procedure of sampling consisted of taking wing membrane biopsies from bats, and was approved by the National Animal Research Authority, East China Normal University (approval ID 20080209). Bats were immediately released *in situ* after tissue sampling. Currently in China no specific permissions are required for sampling bats.

### DNA extraction, amplification, cloning and sequencing

**Figure 2:**
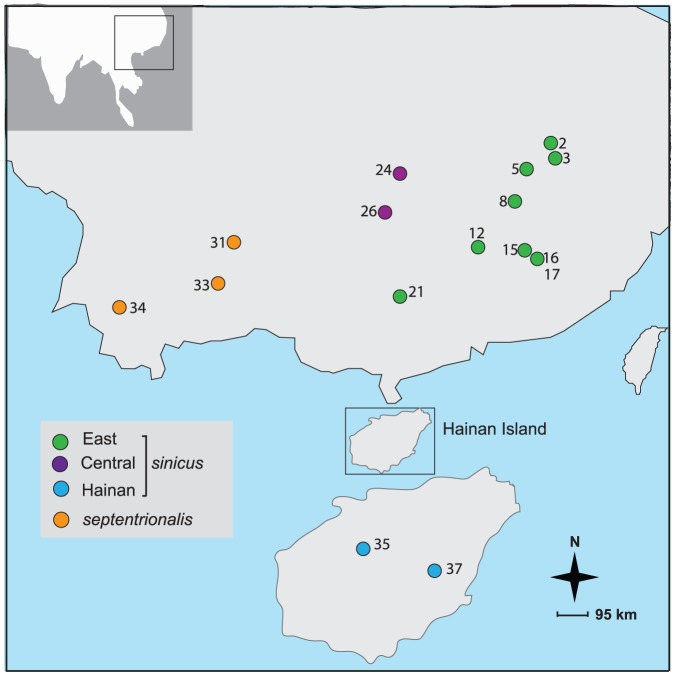
Map showing the sampling sites in this study modified from Mao *et al.* (2013b). Populations are coloured by taxon membership: East *sinicus* (green), Central *sinicus* (orange), Hainan *sinicus* (blue), and *septentrionalis* (purple).

We studied eighteen individuals of *R. sinicus* that were collected as part of a larger study from 15 localities across the Chinese mainland and the offshore Hainan Island (Figure 2). Genomic DNA was extracted using DNeasy kits (Qiagen) and stored at −20°C. We amplified the part of the Central domain and the whole CSB domain of the mitochondrial control region (Figure 1) using the universal primer L16517A (5′-CATCTGGTTCTTACTTCAGG-3′) [35] and the bat-specific primer sH651 (5′-AAGGCTAGGACCAAACCT-3′) [36]. Polymerase chain reactions (PCRs) were carried out in 50 µl volumes (containing 10–50 ng DNA, 0.25 mM of each primer and 1.0 U Taq) on a PTC-220 thermal cycler (Bio-Rad). The thermal profile was 95°C 5 min; 34 cycles of 30 s at 94°C, 30 s at 50°C, 40 s at 72°C; 72°C for 10 min. For each individual, PCR products contained one or two different fragment sizes. If two fragments presented, the large and small ones were coded as individual ID_L and individual ID_S, respectively. Each fragment was cloned using pGEM-T Easy vector (Promega). For each of the two fragment sizes, 8–12 clones were picked and sequenced with both M13 primers on an ABI PRISM 3700 automated sequencer (Applied Biosystems). To check for the presence of the ambiguous sites in numts, the small fragment was also directly sequenced using the PCR primers. Finally, long-range PCRs were conducted for five individuals using primers Long-F (5′-CTAATACCACTCGCAAGC-3′) and Long-R (5′-TCCATAGGGTCTTCTCGT-3′), whose products encompass cytochrome b, control region and 16SRNA with the length of around 4500 bp. The thermal profile for long-range PCRs was 94°C 60 s; 35 cycles of 30 s at 94°C, 30 s at 48.8°C, 4.5 min at 72°C; 72°C for 10 min.

Sequences were aligned using CLUSTAL_X 1.83 [37] in MEGA v.5.0 [38] and edited by eye. All sequences generated in this study were deposited in GenBank (accession numbers: KF994647–KF994769). The full-length control region sequences from three *R. sinicus* (accession number: DQ642887-89), seven other congeneric species (accession numbers: DQ642890, DQ642894, DQ642895, DQ642897, EU053156, EU053159, EF217358) and three species from the related genus *Hipposideros* (accession numbers: EU053164, JX861077, JX861075) were also included in the phylogenetic analysis. The final alignment of the control region sequences did not include the stretch of R2 repeats that prevented alignment.

### Phylogenetic analysis

To investigate the number and timing of transfer events from the mitochondrial to nuclear genome during the evolutionary history of *R. sinicus*, we undertook phylogenetic reconstructions using Bayesian Inference (BI) in MrBayes 3.1.2 [39]. MODELTEST 3.0 [40] and the hierarchical likelihood ratio tests (hLRTs) were used to select the best fitted substitution model for the datasets of all sequences as HKY+I+G [I = 0.2151; G = 0.8264]. In order to test whether the mode of evolution differs between the authentic mtDNA and the numts, MODELTEST was also performed for the datasets of the authentic mtDNA and the numts separately (the mtDNA: TrN+I+G [I = 0.6010; G = 0.4166]; the numts: HKY). For BI, we performed two simultaneous Metropolis-coupled Markov chain Monte Carlo runs, each comprising four chains and 10 million generations. Trees and parameters were sampled every 100 generations, and the first 25% of the sampled trees were discarded as a burn-in. To visually illustrate the relationships among haplotypes from each of the three clades in the phylogenetic trees (see Results), we constructed statistical parsimony networks in the package TCS version 1.21 [41]. Finally, the net sequence divergence [42] among the three clades was calculated using Kimura 2-parameter (K2P) implemented in MEGA v.5.0. For comparisons with the sequence divergence from between inter-species, authentic mtDNA sequence from an outgroup species (i.e. *R. affinis*) was also included.

## Results

### Intra-individual variations in the mitochondrial control region

**Figure 3:**
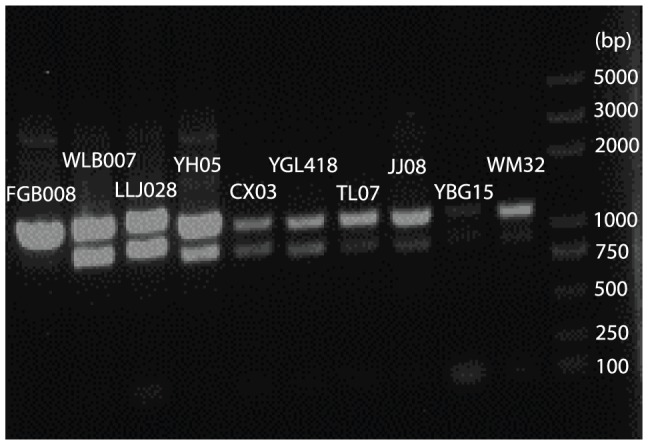
Size variation observed in PCR products for representative individuals from each of the four *R. sinicus* taxa. FGB008, WLB007, LLJ028, and YH05 were from East *sinicus*; CX03 and YGL418 were from Hainan *sinicus*; TL07 and JJ08 were from Central *sinicus*; YBG15 and WM32 were from *septentrionalis*.

The PCR products for almost all individuals contained two fragment sizes (Figure 3), suggesting the occurrence of intra-individual variation in the mitochondrial DNA (mtDNA). At least two factors could cause this pattern: nuclear mitochondrial copies (numts) and heteroplasmy. Long-range PCRs indirectly supported the presence of numts by showing only one segment for each individual (see example in Figure S1). In total, 123 sequences were generated from 18 individuals (seven from East *sinicus*, four from Hainan *sinicus*, three from Central *sinicus*, and four from *septentrionalis*). Due to the failure of cloning and/or sequencing, some individuals (FGB008, QF03, JJ09, SHC009 and WM32) only contained sequences from either the large or small fragment.

*Numts* Results from the direct sequencing after PCR revealed that over half of the individuals exhibited ambiguous sequences with more than two double peaks in their chromatograms (see examples in Figure S2), suggesting the occurrence of numts or contamination of numts in authentic mtDNA sequences. By cloning, numts sequences were identified from both small and large fragments, and were recognized on the basis of several characteristics (see details in Discussion). No tandemly repeated sequences (R2 in Figure 1) were detected in all numts sequences.

*Heteroplasmy* All authentic mitochondrial sequences were from the large-sized fragments except for one individual (ASY20) and exhibited both sequence and length variation. Heteroplasmic sequence variants differed in their R2 repeats as well as in other parts of the control region. In total R2 repeats were found to be composed of five 11-bp long motifs that differed from each other by one or two base pairs (motif A: AACGTACACGT; B: GACGTACACGT; C: AACGTATACGC; D: AACGCATACGC; E: AACACATACGC).

**Figure 4:**
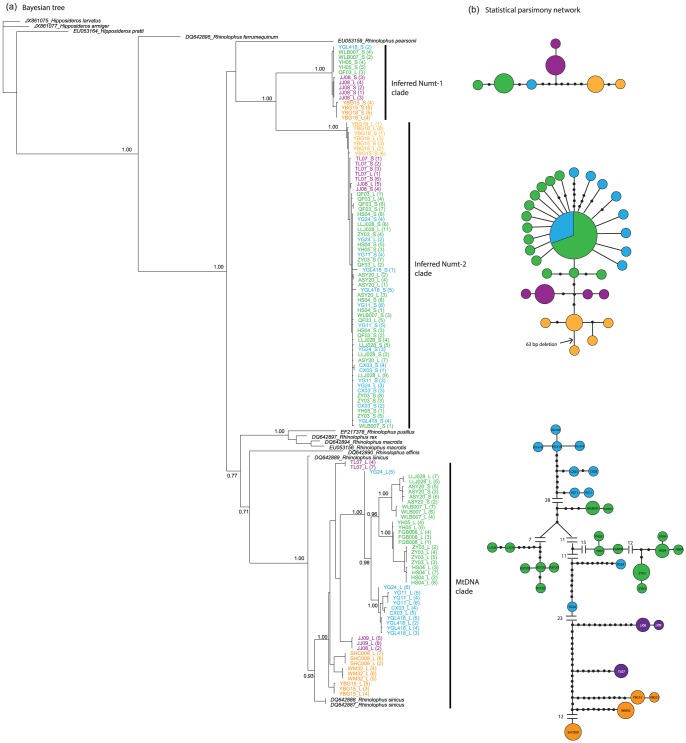
Trees and networks based on the control region sequences excluding the R2 repeats. (a) Phylogenetic tree constructed using Bayesian inference. Numbers on branches are posterior probabilities. Individuals are coded as ID_L or ID_S (the clone number); (b) Statistical parsimony networks for each clade. Each circle represents a single haplotype and circle size is scaled by haplotype frequency. Filled black circles represent missing or unsampled haplotypes. The numbers between haplotypes and sub-networks represent the mutational steps between them. Individuals and haplotypes are coloured by taxon membership as in Figure 1.

We also found phylogeographic structure in the type and number of repeats. For individuals from East *sinicus* and Hainan *sinicus*, the R2 repeats exhibited a complex structure with the combination of two or three different repeat motifs. In contrast, the structure of the R2 repeats in Central *sinicus* and *septentrionalis* individuals was simple with only one repeat motif except for one individual from *septentrionalis* (Table 1). Likewise, individuals from East *sinicus* and Hainan *sinicus* showed heteroplasmy in the sequences of the control region excluding the R2 repeats with more than one haplotype for each individual, whereas individuals from Central *sinicus* and *septentrionalis* did not show heteroplasmy with only one haplotype for each individual (except for one from *septentrionalis* YBG15, see the haplotype network of the mtDNA clade in Figure 4b). Heteroplasmic length variation due to tandemly repeated sequences of the R2 in the CSB domain was also observed in all individuals of *R. sinicus*. The number of the repeated motifs in these sequences ranged from 14 to 20 except for one from Central *sinicus* and one from *septentrionalis* showing only 7 and 3 repeated motifs, respectively (Table 1).

**Table 1:** Heteroplasmic sequence and length variations of the R2 repeated sequences in the mitochondrial control region.

Taxa	ID	Size class	length	N	Repeat motifs
East *sinicus*	FGB008	S1	220	20	(A)_4_(B)_1_(A)_5_(B)_2_(A)_6_(B)_2_
		S2	220	20	(A)_4_(B)_1_ (A)_5_(B)_2_(A)_7_(B)_1_
	ZY03	S1	209	19	(A)_1_(B)_1_(A)_5_(B)_2_(A)_3_(B)_1_(A)_3_(B)_3_
		S2	198	18	(A)_1_(B)_1_(A)_5_(B)_2_(A)_2_(B)_1_(A)_3_(B)_3_
		S3	187	17	(A)_1_(B)_1_(A)_4_(B)_2_(A)_2_(B)_1_(A)_3_(B)_3_
		S4	187	17	(A)_1_(B)_1_(A)_5_(B)_1_(A)_2_(B)_1_(A)_3_(B)_3_
	YH05	S1	187	17	(A)_10_(B)_7_
	HS04	S1	176	16	(A)_2_(B)_7_(A)_3_(B)_4_
		S2	154	14	(A)_2_(B)_2_(A)_2_(B)_2_(A)_1_(B)_5_
		S3	143	13	(A)_2_(B)_4_(A)_3_(B)_4_
	LLJ028	S1	209	19	(C)_1_(D)_6_(C)_1_(D)_4_(C)_5_(D)_1_(C)_1_(D)_1_(C)_1_
		S2	198	18	(C)_2_(D)_4_(C)_1_(D)_4_(C)_5_(D)_1_(C)_1_(D)_1_(C)_1_
	WLB007	S1	209	19	(D)_1_(C)_1_(D)_1_(C)_1_(D)_4_(C)_2_(D)_1_(C)_3_(D)_3_(C)_2_
		S2	209	19	(D)_1_(C)_1_(D)_1_(C)_1_(D)_3_(C)_3_(D)_1_(C)_3_(D)_3_(C)_2_
	ASY20	S1	209	19	(E)_2_(D)_1_(E)_3_(D)_1_(E)_3_(D)_1_(E)_1_(D)_1_(E)_1_(C)_1_(D)_1_(C)_4_(D)_1_(C)_2_
		S2	198	18	(E)_1_(D)_1_(E)_1_(D)_5_(E)_1_(C)_1_(D)_1_(C)_4_(D)_1_(C)_2_
		S3	176	16	(E)_1_(D)_5_(E)_1_(C)_1_(D)_1_(C)_4_(D)_1_(C)_2_
		S4	165	15	(E)_1_(D)_1_(E)_1_(D)_4_(E)_1_(C)_1_(D)_1_(C)_2_(D)_1_(C)_2_
Hainan *sinicus*	YGL418	S1	214	19	(C)_1_ AACGC(D)_6_(C)_1_(D)_1_(C)_3_(D)_2_(C)_1_(D)_1_(C)_3_
		S2	203	18	(C)_1_ AACGC(D)_5_(C)_1_(D)_1_(C)_3_(D)_2_(C)_1_(D)_1_(C)_3_
		S3	181	16	(C)_1_ AACGC(D)_4_(C)_1_(D)_1_(C)_3_(D)_1_(C)_1_(D)_1_(C)_3_
	YG24	S1	209	19	(C)_3_(D)_3_(C)_1_(D)_1_(C)_1_(D)_1_(C)_1_(D)_3_(C)_1_(D)_1_(C)_1_(D)_1_(C)_1_
	YG11	S1	176	16	(C)_5_(D)_1_(C)_4_(D)_1_(C)_1_(D)_1_(C)_1_(D)_1_(C)_1_
		S2	154	14	(C)_3_(D)_1_(C)_4_(D)_1_(C)_1_(D)_1_(C)_1_(D)_1_(C)_1_
	CX03	S1	198	18	(C)_7_(D)_3_(C)_4_(D)_1_(C)_3_
		S2	187	17	(C)_6_(D)_3_(C)_4_(D)_1_(C)_3_
Central *sinicus*	TL07	S1	209	19	(C)_19_
		S2	176	16	(C)_16_
	JJ09	S1	198	18	(C)_18_
	JJ08	S1	77	7	(C)_7_
*septentrionalis*	SHC009	S1	209	19	(C)_10_(D)_1_(C)_8_
		S2	187	17	(C)_17_
	WM32	S1	209	19	(C)_19_
		S2	198	18	(C)_18_
	YBG15	S1	165	15	(C)_15_
		S2	33	3	(C)_3_

Size class means classes of sequences with different length and/or motifs. Repeat motif A: AACGTACACGT; B: GACGTACACGT; C: AACGTATACGC; D: AACGCATACGC; E: AACACATACGC. N is the total number of tandem repeats.

### Phylogenetic analysis

The alignment of 123 sequences spanned 724 bp and contained 131 indels. Phylogenetic analysis using the BI method revealed three highly supported clades, two of which were from numt sequences (hereafter called Numt-1 clade and Numt-2 clade), and the third was from mtDNA sequences (hereafter called mtDNA clade) (Figure 4a). The two numt clades exhibited extreme net sequence divergence with their assumed mitochondrial counterparts (Numt-1 vs mtDNA clade, 18.4%; Numt-2 vs mtDNA clade, 21.9%) comparing with the sequence divergence of authentic mtDNA between species (*R. sinicus* vs *R. affinis*, 20.8%).

Each of the Numt clades (Numt-1 and 2) contained sequences from all four taxa of *R. sinicus* although the Numt-2 clade contained more cloned sequences than did the Numt-1 clade (61 versus 15 respectively). The average sequence divergence between the Numt-1 and Numt-2 clade was 16.8%. Consistent with the observation of very short branches in the trees, few mutational steps were detected among the four *sinicus* taxa in the haplotype network of each Numt clade (Figure 4b).

In the mtDNA clade, phylogenetic relationships among the four *R. sinicus* taxa were similar to those previously recovered based on two mitochondrial protein coding genes and four nuclear genes [34] although both central *sinicus* and *septentrionalis* were paraphyletic in this study; East *sinicus* and Hainan *sinicus* clustered together, and Central *sinicus* showed a closer relationship with *septentrionalis* than with the other two *sinicus* taxa (Figure 4a). These relationships were clearly displayed in the haplotype network (Figure 4b). In comparison with the above two Numt clades, more mutational steps were observed among the four *R. sinicus* taxa in the network of the mtDNA clade, indicating that the mutation rate was substantially reduced after mitochondrial fragments were transferred into the nuclear genome.

## Discussion

Although numts and mitochondrial heteroplasmy have been recorded in multiple species groups [4], [6], [43], few studies have investigated their patterns at the intra-specific level. Here we reported the presence of both numts and heteroplasmy in the mitochondrial control region of the Chinese horseshoe bat *Rhinolophus sinicus*. In addition, we conducted an assessment of the impact of numts and heteroplasmy on phylogenetic and phylogeographic reconstructions, which might be pertinent to many mtDNA-based studies where these phenomena were not recognized and accounted for.

### The occurrence of numts and heteroplasmy in the mitochondrial control region

*Numts* In this study numts were recognized and distinguished from heteroplasmy based on at least three sources of evidence. First, all numts sequences did not contain tandemly repeated sequences that were considered to affect the efficiency of mtDNA genome replication [25]. Second, in the phylogenetic trees, all numts were classified together and fell outside the monophyly of authentic mtDNA sequences. In addition, with the exception of *R. pearsoni*, these numts did not cluster with the full-length control region sequences of three *R. sinicus* and the outgroups (Figure 4a), all of which were generated using the long-range PCR and were confidently treated as authentic mitochondrial sequences. Phylogenetic analysis has been proven to be a good method to distinguish numts from native mitochondrial sequences in many studies [44]. Third, consistent with the view that numts are considered to have reduced mutation rates relative to their mitochondrial counterparts [45], we found that fewer mutational steps had occurred among the four *R. sinicus* taxa in both Numt-1 and Numt-2 clades than in the mtDNA clade (Figure 4b).

Our results add to a small number of studies reporting numts in bats, and suggest that translocations of the mitochondrial DNA into the nucleus may have occurred recurrently in the same species group. In particular, one or two translocations appear to have occurred before the diversification of *R. sinicus*, leading to two divergent clades (i.e. Numt-1 and Numt-2 clade) that have also both diverged from the mitochondrial sequence. At least three scenarios can be considered to explain the origin of the Numt-1 and Numt-2 clades in this study. First, they might have originated from the same taxon but at two different time points. Remarkably we also found that the two Numt clades fell outside of the mitochondrial clades of four other congeneric horseshoe bat species from Asia (*R. pusillus*, *R. rex*, *R. macrotis* and *R. affinis*), suggesting that the inferred translocations are ancient events involving the common ancestor of several unrelated horseshoe species, previously inferred to have a common ancestor at 12 MYA [46]. Based on this scenario we predict that numts of the control region will also be present in these and other congeneric horseshoe species, a hypothesis that we are now testing.

Interestingly, we found that more cloned sequences were classified as belonging to the Numt-2 clade than the Numt-1 clade, which could have arisen for a number of reasons. First, this might reflect greater amplification success for Numt-2 sequences; for example, if the Numt-2 clade was older than the Numt-1 clade, then sequences of the Numt-2 clade may be closer to the current universal primer sequence. Alternatively the greater number of cloned sequences from the Numt-2 clade could have resulted if this sequence occurs as multiple copies in the nuclear genome, due to several integrations [47] and/or duplication events after the original translocation. A third possibility is that these two Numt clades might result from translocations to different genomic regions that exhibit different modes of evolution and, therefore, diverge with different rates following the translocation events. However, this scenario was not supported by a MODELTEST analysis, which estimated the same substitution model (HKY) for the two inferred numt clades. Finally, we cannot rule out the possibility that the Numt-1 and Numt-2 clades originated at similar times but from different taxa. Unfortunately, based on the current low coverage of the *Rhinolophus* taxa, we have no way to establish which species or common ancestor is the source of the Numt-1 or Numt-2 clade. In the future phylogenetic analysis based on the control region sequences from other *Rhinolophus* can be used to determine the source taxa for the two Numt clades.

*Heteroplasmy* In this study heteroplasmy occurred as both sequence and length variation of the mitochondrial control region. Although heteroplasmic length variation due to tandemly repeated sequences within the mitochondrial control region has been reported in many bat species [19], [20], [31], [32], few studies have explored the structural variations of the tandemly repeated sequences at an intra-specific level. In this study, the unit length of the R2 repeated sequences (i.e. repeated motifs) was 11 bp, which was within the range of previously reported lengths of less than 10 bp to over 200 bp [48]. This 11 bp motif also appeared in other six congeneric species [20] although the sequences were different among them, suggesting that the 11 bp motif might be established early in the common ancestor of this genus.

Within *R. sinicus*, five different repeated motifs were detected; however, four of them appeared only in East *sinicus* and Hainan *sinicus*, and Central *sinicus* and *septentrionalis* exhibited only one motif except for one individual of *septentrionalis*. This structural variation suggests that the Central *sinicus* is more closely related to *septentrionalis* than to the other two *sinicus* taxa, a finding that is consistent with previous results based on two mtDNA protein coding genes [34]. The shared absence of repeated motifs in Central *sinicus* and *septentrionalis* could arise from introgression of the complete mitochondrial genome from *septentrionalis* to Central *sinicus*, as suggested previously [33], [34]. Alternatively, this simple structure could suggest that the Central *sinicus* and *septentrionalis* represent the ancestral form before *R. sinicus* diverged into several taxa, although why this has been retained whereas more complex repeated sequences have evolved in East *sinicus* and Hainan *sinicus*, is not known. Further analysis of the structure of the repeated sequences from *R. thomasi*, a species closely related to *R. sinicus*, will be needed to test this assumption.

Heteroplasmic sequence variation outside of the R2 repeats can result from mutations in the female line that are vertically transmitted [30]. Consistent with this explanation, most individuals examined in this study showed sequence variation at only one or two positions. Alternatively, the observed heteroplasmy might be caused by errors introduced by *Taq* polymerase errors during PCR cloning. If the *Taq* error rate is assumed to be 7.2×10^−5^ per bp per cycle [49], we would expect to have 1.77 errors in each sequence based on the length of the sequence (724 bp) and the number of cycles used for PCR (34 cycles). Taking this error rate into account, we can thus expect 26% of clones to have >2 errors and 10% to have >3 errors, but only 3% to have >4 errors. Theoretically, therefore, at least a fraction of the variation seen within numt clades may represent artefacts due to PCRs and cloning errors. However, an experimental cross-check of the number of errors introduced by amplification and cloning suggested that the error rate might be rather lower than these calculations suggest. Specifically, we repeated PCR amplification for one individual (FGB008), and cloned and sequenced one fragment. This cloned fragment was then re-cloned and 26 positive clones sequenced. Based on this test, a total of 23 mutations were detected across 26 clones, with an average of 0.9 change per clone, and 8 clones (∼30%) were identical. Finally, heteroplasmic sequence variation could have resulted from paternal inheritance of mtDNA, as has been reported in a range of taxa [50], [51], [52]. One haplotype of individual (YG24) fell outside the whole [East *sinicus*+Hainan *sinicus*] clade in the phylogenetic trees and network, and shared several polymorphisms with [Central *sinicus*+*septentrionalis*]. This extreme sequence divergence between two haplotypes of this individual could not be explained by the above scenarios; perhaps a more likely explanation is that hybridization occurred between [East *sinicus*+Hainan *sinicus*] and [Central *sinicus*+*septentrionalis*], followed by paternal mtDNA leakage into the former from the latter [53]. Nonetheless, except for humans, paternal mtDNA leakage has been rarely described in mammals and appears to occur at extremely low rates [54]. Additional samples will need to be analyzed using allele-specific real-time quantitative PCR to determine the frequency of parental leakage in *R. sinicus* [51].

### Implications for future mtDNA phylogenetic studies

Relatively few phylogenetic and phylogeographic studies adequately consider and test for the presence of numts and heteroplasmy, in spite of the problems these phenomena may present. Heteroplasmy can cause phylogenetic problems if it arose as a result of parental leakage of mtDNA from other divergent lineages due to hybridization [55]. Even when heteroplasmy resulted from somatic mutations, it still could lead to erroneous inferences of demographic history by generating high levels of polymorphism in populations. Fortunately, heteroplasmy - especially due to length variation - is commonly restricted to the mitochondrial control region [14], [15], [16], [56] and, therefore, studies may avoid this issue by instead focusing on mtDNA protein-coding genes.

Numts, on the other hand, are less easy to address. Those that originated recently may be scattered throughout the phylogenetic tree, leading to erroneous conclusions about population history and structure [57]. In our study numts did not cause problems in the phylogenetic analysis because they all formed a separate clade from mtDNA sequences, probably due to their ancient origin. However, had these numts been unrecognized and included in the mtDNA-based analysis, they would have potentially led to the mistaken recognition of two distinct species with the current *R. sinicus*. Currently, approaches proposed to detect and avoid contamination by numts during PCR of mtDNA sequences include not using universal primers [9], using long-range PCR or reverse transcription PCR to obtain real mtDNA sequence for primer design, the use of pre-PCR dilution [10], and the avoidance of non-coding segments such as the control region [11]. On the other hand, once recognized or reliably distinguished from authentic mtDNA, numts can be very informative in evolutionary biology, being useful molecular fossils for phylogenetic comparisons [28], [45], [58], dating divergence between clades [57] and reconstructing past evolutionary history of divergent lineages in the secondary contact zone [55]. Recent next-generation sequencing (NGS) technologies have the potential to renew interest in numts by generating the complete mitochondrial genome sequences rapidly and cheaply, and allowing the identification of numt genomic locations.

## Supporting Information

Figure S1 **Size view of the long-range PCR products for five representative individuals.**
(EPS)

Figure S2 **The chromatograms of direct PCR sequencing for four representative individuals.** Ambiguous sites with double peaks were shown in red square.
(EPS)
